# Effect of yoga relaxation techniques on performance of digit–letter substitution task by teenagers

**DOI:** 10.4103/0973-6131.43293

**Published:** 2009

**Authors:** Balaram Pradhan, H R Nagendra

**Affiliations:** Division of Yoga and Life Sciences, Swami Vivekananda Yoga Anusandhana Samsthana, Bangalore, India

**Keywords:** DLST, yoga, relaxation, meditation

## Abstract

**Background/Aims:**

Memory and selective attention are important skills for academic and professional performance. Techniques to improve these skills are not taught either in education or company training courses. Any system which can systematically improve these skills will be of value in schools, universities, and workplaces. Aims:To investigate possible improvements in memory and selective attention, as measured by the Digit–Letter Substitution Task (DLST), due to practice of Cyclic Meditation (CM), a yoga relaxation technique, as compared to Supine Rest (SR).

**Materials and Methods:**

Subjects consisted of 253 school students, 156 boys, 97 girls, in the age range 13–16 years, who were attending a 10-day yoga training course during summer vacation. The selected subjects had English as their medium of instruction in school and they acted as their own controls. They were allocated to two groups, and tested on the DLST, immediately before and after 22.5 minutes practice of CM on one day, and immediately before and after an equal period of SR on the other day. The first group performed CM on day 9 and SR on day 10. For the second group, the order was reversed.

**Results:**

Within each group pre-post test differences were significant for both the relaxation techniques. The magnitude of net score improvement was greater after SR (7.85%) compared to CM (3.95%). Significance levels were *P* < 0.4 × 10^-9^for SR and *P* < 0.1 × 10^-3^ for CM. The number of wrong attempts also increased significantly on both interventions, even after removing two outlier data points on day 1 in the SR group.

**Conclusions:**

Both CM and SR lead to improvement in performance on the DLST. However, these relaxation techniques lead to more wrong cancellation errors.

## INTRODUCTION

Over the past thirty years or more, yoga and associated meditation techniques have come to be accepted as important means of reducing effects of stress.[1] A wide variety of studies in these fields have been done,[2–4] and in one of them different techniques such as progressive muscular relaxation, biofeedback, relaxation, and mental imagery were compared by meta-analysis.[5] One study concluded that different techniques produce different spectrum of effect sizes for different tasks.[6] The early hypothesis by Benson[7] that there is a universal ‘relaxation response’ that is same for any technique has been convincingly refuted.[8] Rather it is recognized that each technique produces effects in specific brain regions, and that precise magnitude of benefits for a particular task depends on the extent to which that brain region is used in task performance.

Today, the most effective of these techniques are well accepted as being of substantial benefit for stress in the workplace and in other aspects of life, and are widely taught as specific coping skills, particularly for professionally incurred stress. These stress-reduction techniques have been found useful in reducing a variety of stress-related symptoms, such as anxiety, neuroticism, depression, and hypertension. For example, ‘Deep Breathing Meditation Exercises’ have been reported to decrease anxiety, nervousness, self-doubt, and concentration loss.[9] They have also been found to improve measures of emotional intelligence.[10] Such techniques are most effective when practiced daily for an extended period of time.[11]

In addition to reduction in stress symptoms, beneficial effects of various relaxation techniques include: feeling of well-being, sense of calmness and relaxation in activity, improved sleep, less emotional reactivity, increased inner directedness (self-awareness), and improved self-care.[12] Improved performance has also been found on a variety of psychological tests, such as IQ,[13] Tower of London Test,[13,14] Baddley Tests of Verbal and Spatial Memory,[15,16] Six Letter Cancellation Test (SLCT),[17] and so on.

In this study, the influence of yoga relaxation techniques on performance of the Digit–Letter Substitution Task (DLST) was investigated. The DLST depends on selective attention and memory. It is easily understood and performed and suitable for subjects of all ages, including school students. It was therefore given to participants in a 10-day personality development camp held for early teenage school students during their summer vacation. The two techniques chosen were the easily performed Cyclic Meditation (CM) developed at sVYASA,[18] and the Supine Rest (SR) position known as ‘sleep posture’ (*shavasana*), generally done at the end of yogasana practice. The reasons are as follows.

*Shavasana* has been found to reduce physiological arousal,[19] and to be effective in helping practitioners cope with stress manifestations, for example, Bera *et al*. found recovery from induced physiological stress was significantly faster for supine posture with additional progressive relaxation, compared to resting, sitting in a chair, or plain *shavasana* (SR).[20] In another study, a significant decrease in breath rate was noted after performance of the yoga-based Isometric Relaxation Technique (IRT), when compared to SR.[21] IRT forms an integral part of the CM, which we now describe in detail.

CM combines ‘stimulating’ and ‘calming’ practices. Such yoga practice is described in the *Mandukya Karika*, a text associated the *Mandukya Upanishad*, which suggests that such a combination is helpful in attaining mental equilibrium. CM consists of the practice of yoga postures (*asanas*) interspersed with periods of relaxation in shavasana. After the period of practice, significant reductions in oxygen consumption occur, compared to an equal period of *shavasana*.[22,23] CM has been found particularly effective in relieving stress, and is widely applied in professional stress management programs.

Recent studies on CM suggest that during the yoga posture phase, predominantly sympathetic activation occurs, whereas after CM, the parasympathetic nervous system becomes dominant.[24] The overall result is a greater reduction in energy expenditure than in SR.[25] CM has also been found to enhance the P300 wave in the evoked potential,[26] a fundamental cognitive process involving attention and immediate memory.[27]

Since the DLST involves memory and selective attention, it was hypothesized that CM would increase performance on the test. For convenience of subject availability, it was decided to study changes on DLST scores after performance of CM, compared to SR, in school students attending one of sVYASA’s smmer vacation 10-day personality development camps.

## MATERIALS AND METHODS

### Subjects

The subjects in the study were school students attending a 10-day yoga training course during their summer vacation. Since the protocol for the DLST is given in English, subjects were selected first, for being between ages 13 and 16; and second, for English being their medium of instruction in school. Exclusion criteria included: first, any history of neurological or psychiatric disturbance; second, use of any medication affecting the central nervous system; third, any history of learning disability; and last, insufficient proficiency in English to understand how to take the test. Twelve further students were eliminated when they made excessive numbers of wrong substitutions on a preliminary test. (Two further subjects were eliminated for similar reasons after the first test, see data analysis.) Of more than 300 students in the correct age range attending the yoga training camp, 253 (156 boys and 97 girls) were selected. The mean age for the group was 13.95 ± 0.99 years [Table 1]. Study participants were given a complete description of the study, following which they were asked to give written informed consent. Since the comparison study protocol required two equal groups with the order of the two interventions reversed, the 253 subjects were allocated to two groups of 133 (80 boys and 53 girls) and 120 (76 boys and 44 girls).

**Table 1:** Age group mean ± standard deviation of study subjects

	Mean ± SD	*n*
Girls	14.02 ± 1.02	97
Boys	13.91 ± 0.97	156
Total	13.95 ± 0.99	253

### Procedure

As participants in the yoga personality development camp, all the subjects were trained in the practice of both CM and SR over the 7-day period preceding the start of the study. Rather than being a random control design, the study was self-controlled, with all study participants being measured before and after a period of CM, and before and after a similar period of SR. The two sets of measurements took place on successive days at the end of the yoga camp, days 9 and 10. In order to allow for any possible learning process that might have resulted from the first day’s tests, the subjects were allocated to two equal-sized groups, with the first group doing CM on day 9 and SR on day 10, and the second group with the order reversed. Subjects were tested on the DLST, immediately before and after a session of CM of 22.5 minutes duration on one day, and immediately before and after an equal period of SR on the other day.

### Instrument

The DLST worksheet consists of a 8 rows × 12 columns array of random digits 1–9. Subjects are seated with the worksheet upside down until the start of the test. They were also given a coding sheet naming the specific letter to substitute for each digit 1–9 in that particular test, the same coding applying to an entire test group. Subjects were instructed to make their own choice of letter substitution strategy, whether horizontally, vertically, or selecting each particular digit randomized in the array one at a time. They were told to substitute as many target digits as possible in the specified time of 90 seconds. Finally, they were instructed to turn over the worksheet and start the test. Each test was timed on a standard stopwatch.

Because the tests were administered with such a short intervening time interval, immediately before and after an intervention of only 22.5 minutes, different worksheets and coding were used for each test, with different digit–letter pairing in the key and differently randomized arrays of digits on the worksheet.

Scoring the DLST counts both the total number of substitutions attempted, and the number of wrong substitutions. Net score is obtained by deducting the latter from the former. Scoring was carried out by persons unaware of when the assessment was made, whether it was ‘before’ or ‘after’ the intervention, or whether the subject was engaged in CM or SR on that day.

The use of this DLST protocol to study immediate effects has already been validated for the Indian population.[28]

### Intervention

Subjects were instructed to keep their eyes closed throughout the time periods of practice of both CM and SR. CM used prerecorded instructions, which emphasized the need to carry out the practice slowly, with awareness and relaxation. The practice starts with subjects lying on their backs in *shavasana* and consists of the following sequence:

- Repetition of a verse from the *Mandukya Upanishad*[29] (0:40 minutes).
- Isometric contraction of the muscles of the body ending with supine rest (1:00 minutes).
- Slowly coming up from the left side and standing at ease (*tadasana*), ‘balancing’ the weight on both feet, called centering (2:00 minutes).
- The first stretching posture, bending to the right (*ardhakatichakrasana*) (1:20 minutes).
- *Tadasana* with instructions about relaxation and awareness (1:10 minutes).
- *Ardhakatichakrasana* bending to the left (1:20 minutes).
- *Tadasana* as previously (1:10 minutes).
- Forward bending (*padahastasana*) (1:20 minutes).
- *Tadasana* as previously (1:10 minutes).
- Backward bending (*ardhachakrasana*) (1:20 minutes).
- Slowly coming down into the supine posture (*shavasana*) with instructions to relax different parts of the body in sequence (10:00 minutes).

All postures are practiced slowly, with instructions to be aware of all felt sensations. Total duration of practice is 22.5 minutes.[18]

During the sessions of SR, subjects lie on their back in sleep posture (*shavasana*) with eyes closed, legs apart, and arms away from the sides of the body. This practice was also given for 22.5 minutes, the same as for CM, timed on a stopwatch.

To allow for any possible learning effect, the two groups received the two interventions in reverse order.

### Data analysis

Statistical analysis was done using SPSS (version 10.0). The Kolmogorov test of normality showed that the total scores and net score data were normally distributed, but that wrong substitutions data were not (Kolmogorov–Smirnov test, *P* < 0.05). Hence, Student’s paired ’ *t* ’ test was used for total and net scores, and the nonparametric Wilcoxon signed ranks test was used for the analysis of wrong substitutions, specifically for within group pre-post comparisons for both CM and SR. The first day SR group contained two outliers with 9 and 10 wrong substitutions, respectively. Since these were over five standard deviations from the mean, they were removed, and the data analyzed without them included.

## RESULTS

Mean values and standard deviation for total scores, wrong substitutions, and net scores on all tests are given in Table 2.

**Table 2:** Mean values and standard deviation for digit–letter substitution task total score, net score, and wrong substitution score

Variables	Sessions			
	Cyclic meditation (*N* = 253)		Supine rest (*N* = 253)	
	Pre	Post	Pre	Post
Total score for substitution	60.13±10.98	62.53±13.2***	57.04±12.24	61.67±12.55***
Score for wrong substitution	0.08±0.29	0.2±0.65**	0.11±0.46	0.24±0.75**
Net score for substitution	59.93±11.03	62.3±13.21***	56.95±12.26	61.42±12.55***

*** *P* < 0.001, student’s paired ‘*t*’ test, posts cores compared with respective prescores,

** *P* < 0.01, Wilcoxon signed ranks test, posts cores compared with respective pre scores

There were significant differences on DLST net scores between sessions for the same group, and between groups for the same session. For the whole group of 253 students, the increase in net score means were 7.85% after SR, and 3.95% after CM; both were highly significant: *P* < 0.4 × 10^-9^ for SR and *P* < 0.1 × 10^-3^ for CM. In addition, both groups made more errors after the interventions, 118.18% after SR and 166.31% after CM, with Wilcoxon signed ranks test significances of *P* < 0.5 × 10^-2^ and *P* < 0.4 × 10^-2^, respectively. The SR wrong substitutions data contained two further outliers, who were removed and the data reanalyzed. Group performance on wrong substitutions data is significant in its implications for the wakeful alertness of subjects after these forms of yoga relaxation. Finally, the scores of the two subgroups were analyzed for the first time they took the test on both days. This revealed very significant learning to have taken place, vindicating the protocol’s use of a reverse order for the delivery of the two interventions to the two subgroups on the two days of testing.

## DISCUSSION

A study by Patil and Telles[30] assessed performance on the related SLCT immediately before and after CM and SR. Protocol design was similar with two subgroups doing both interventions in opposite order. Net scores were significantly higher after both practices, though the magnitude was more after CM than SR (24.9 vs 13.6%). Wrong cancellation scores decreased after CM, but not after SR - controls showed no change. CM seems to improves some of the skills used in SLCT performance – selective attention, concentration, visual scanning abilities, and repetitive motor response.[16] This suggests that DLST performance should also improve after performing both kinds of relaxation.

It was therefore not unexpected that DLST performance should improve immediately after both types of yoga-based relaxation sessions. However, results were different from SLCT: in contrast, for DLST, SR sessions produced better performance than did CM. Pre-post improvements on net scores were almost twice as much for SR as for CM (7.85 vs 3.95%).

In addition, the changes in wrong substitution scores after CM and SR suggest that subjects may have become drowsy by the end of the intervention period.

The differences almost certainly arose because the DLST depends on different components of psychomotor performance from the SLCT, namely: (a) sensory information processing ability; (b) central integration of learning and memory, and (c) motor function and coordination.[31] The DLST was developed from Digit Symbol Substitution Test (DSST), one of the subsets of the Wechsler intelligence scale.[32] Substitution tests are essentially speed-dependent tasks that require the subject to match particular signs – symbols, digits, or letters - to other signs within a specified time period. The DLST has the advantage of using letters and digits, signs that are already well-known to those taking the test.[33] Thus, there is no question of a need to learn new symbols while being tested. Such learning ability is definitely not one of the aptitudes on trial. For this reason, the DLST was used instead of the DSST.[34] Substitution tasks involve visual scanning, mental flexibility, sustained attention, psychomotor speed, and speed of information processing.[35,36] Our finding, that both CM and SR enhance task performance, suggests that one of more of these skills is being improved, probably sustained attention.

Previous results also show that CM practice reduces physiological arousal (decreases in oxygen consumption and minute ventilation are observed),[22,23] increases parasympathetic dominance,[24] and decreases energy expenditure.[25] These changes occur together with decreased latency and increased amplitude in the P300.[26] P300 event-related potentials (EPR) reflect fundamental cognitive events requiring attention and immediate memory processes.[27] They also reflect cognitive brain functions like sequential information processing, stimulus discrimination, and short-term memory.[37] Increases in EPR amplitude with attention suggest greater cognitive processing capacity.[38] Neuropsychological tests assessing how rapidly attentional resources are allocated for memory processing[39,40] associate shorter EPR latency with improved cognitive performance. Altogether, these results suggest that CM reduces physiological arousal, simultaneously improving performance on tasks requiring attention. Further studies are required to understand which mechanisms improve task performance. For example, anxiety is known to affect performance on tasks requiring attention.[41] Anxiety reduction during CM and SR practice may have contributed in some way to the observed improvements in performance. The effects of varying age and gender, and lengthening training program duration could also be investigated.
